# Occipital Bone Defect With Meningoencephalocele and Plexiform Neurofibroma in Neurofibromatosis-1

**DOI:** 10.7759/cureus.93773

**Published:** 2025-10-03

**Authors:** Ankita Pandey, Raghavendra Kamalesh, Ankur Patel, Rajesh Malik, E Jayashankar

**Affiliations:** 1 Radiodiagnosis, All India Institute of Medical Sciences, Bhopal, Bhopal, IND; 2 Pathology and Lab Medicine, All India Institute of Medical Sciences, Bhopal, Bhopal, IND

**Keywords:** calvarial dysplasia, meningoencephalocele, neurofibromatosis type 1, occipital bone defect, plexiform neurofibroma

## Abstract

Neurofibromatosis type 1 (NF-1) is the most common phakomatosis with autosomal dominant inheritance, caused by mutations in the NF-1 gene on chromosome 17q11.2, which codes for neurofibromin protein, a negative regulator of the RAS/MAPK pathway. A defect in this can lead to altered cellular growth and tumor development. NF-1 is a complex multi-system neurocutaneous disorder with some of the common manifestations being café au lait macules, axillary/inguinal freckling, and neurofibromas. Musculoskeletal manifestations include scoliosis, sphenoid wing dysplasia, tibial pseudoarthrosis, and, rarely, calvarial defects resulting from dysplastic bone. Central nervous system manifestations range from focal areas of signal intensities (FASIs), optic pathway gliomas, dural ectasia, and meningoceles. We report the case of a 24-year-old male who presented with a progressively enlarging swelling over the posterior scalp and left side of the neck since childhood, which on examination led to suspicion of underlying calvarial defect, and along with general examination findings of multiple cafe-au-lait macules and cutaneous neurofibromas fulfilled the diagnostic criteria for NF-1. Examination revealed a palpable calvarial defect. Cross-sectional imaging (MRI and CT) of the occipital swelling revealed a large plexiform neurofibroma, associated with a bony defect involving the occipital bone and lambdoid suture with herniation of dysplastic posterior fossa structures into the neurofibroma in the form of meningoencephalocele. Our case not only highlights the rare occurrence of neurofibroma associated with rare location of calvarial defect and underlying meningoencephalocele, it identifies the role of imaging in evaluating the extent and characterization of the neurofibromas for management and surveillance.

## Introduction

Neurofibromatosis type 1 (NF-1) is the most common phakomatosis with an autosomal dominant inheritance pattern. Its incidence is approximately one in 2500 to 3000 live births. The affected gene, NF-1, located on chromosome 17q11.2, causes a defective encoding of neurofibromin, a tumor suppressor protein. This leads to unchecked activation of the RAS/MAPK pathway (RASopathy), resulting in altered cellular growth progressing to tumor formation [1,2]. It presents as a complex multi-system disorder having a wide range of manifestations, affecting the skin (café au lait spots, axillary and inguinal freckling, pigmented iris hamartomas), the central nervous system (neurofibromas involving spinal nerves, plexiform neurofibromas (PNFs), non-neoplastic hamartomas of the white matter and basal ganglia, gliomas or astrocytomas affecting the optic-chiasmatic-hypothalamic pathway and brainstem), and the skeletal system [1,2].

Skeletal manifestations often include scoliosis, long bone dysplasia, tibial pseudoarthrosis, short stature, and skull abnormalities such as macrocephaly, hydrocephalus, and calvarial bone dysplasia. The latter most commonly affects the sphenoid wing, orbit, frontal, parietal bones, and sagittal suture, often with associated meningoencephaloceles. A key diagnostic feature of NF-1 is a distinctive osseous lesion such as sphenoid dysplasia, pseudoarthrosis, or anterolateral bowing of the tibia [1,2].

Although calvarial dysplasias are a distinctive feature of NF-1 and often seen involving the sphenoid wing, they rarely involve the occipital bone and lambdoid suture, especially as a single large defect. Only limited case reports and literature are available on this topic to the best of our knowledge [1,3-7].

Here we report a rare case of a 24-year-old male with a large plexiform neurofibroma in the occipital region, accompanied by a bony defect involving the occipital bone and lambdoid suture, with underlying meningoencephalocele. A brief discussion and review of the literature on the calvarial manifestations of NF-1 and its pathogenesis are also provided.

## Case presentation

Clinical presentation

**Figure 1:**
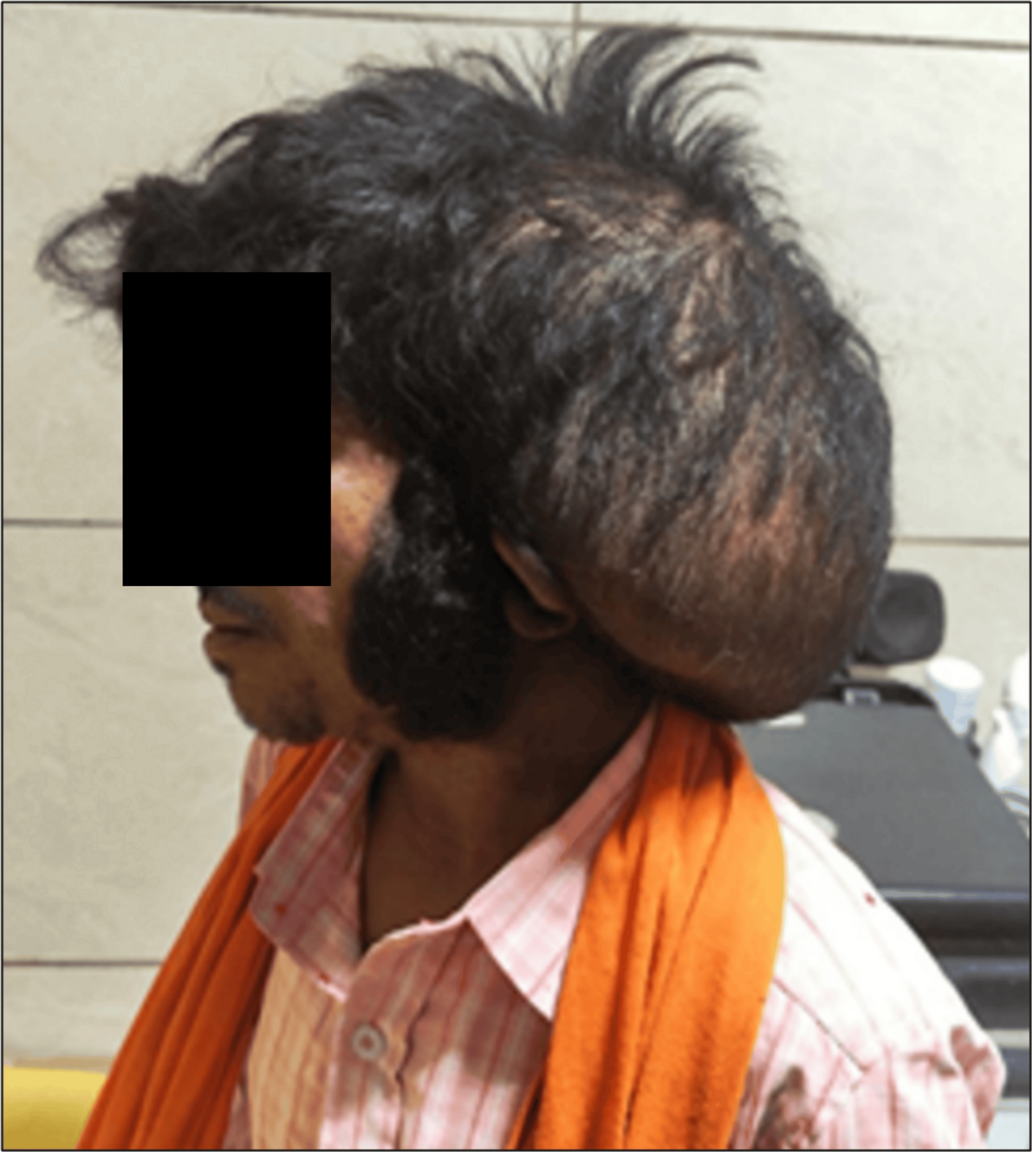
Large swelling on the scalp in the left posterior parieto-occipital region and neck, with overlying hair.

**Figure 2:**
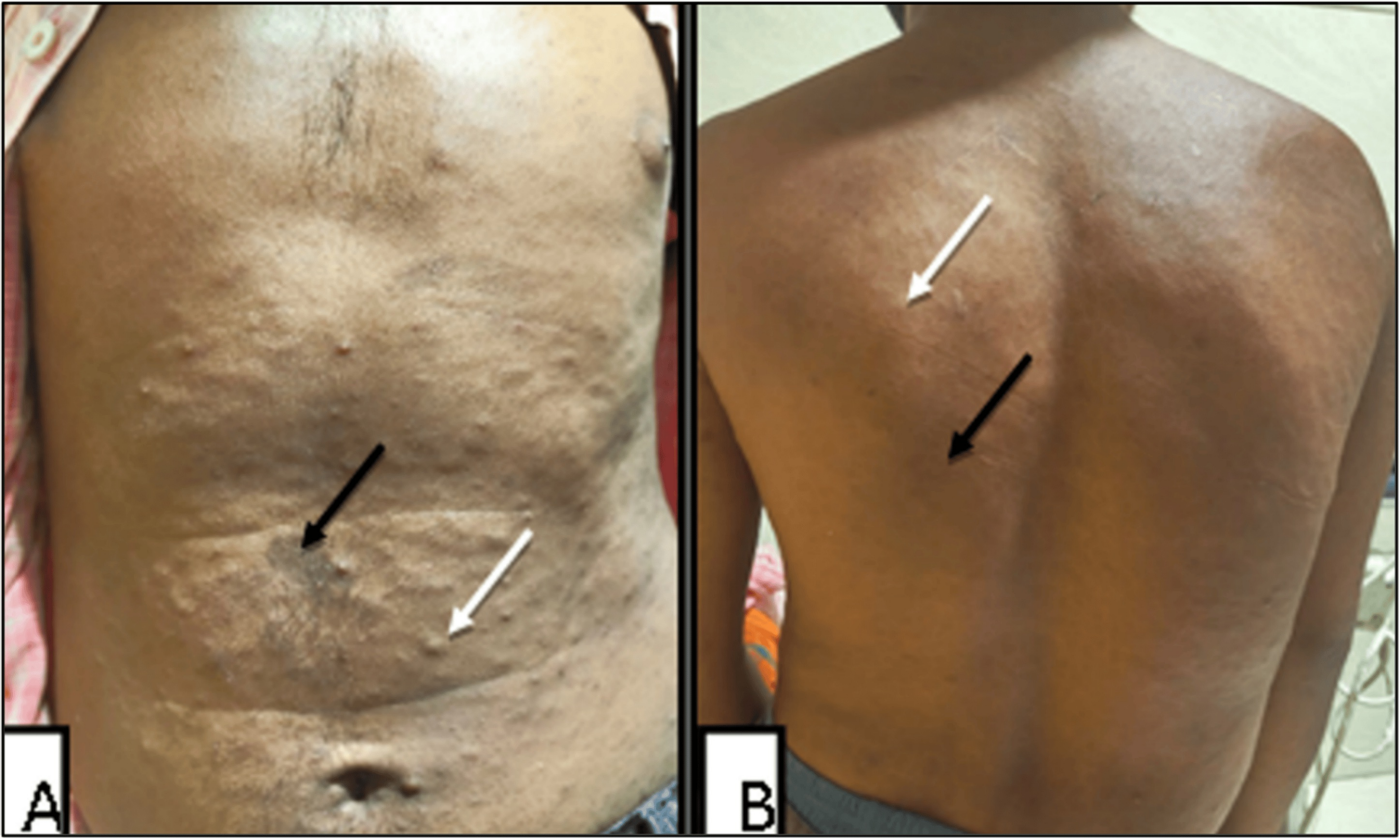
Multiple café-au-lait macules (black arrows) and multiple cutaneous neurofibromas (white arrows) over the anterior (A) and posterior (B) aspect of chest and abdomen.

A 24-year-old male presented to the plastic surgery outpatient department with a large swelling on the posterior scalp in the occipital region and on the left side of the neck. The swelling has been present since childhood and has gradually increased in size. The patient also vaguely narrates a remote history of a fracture in the right leg (no documentation available); however, he has no residual disability in the right leg. No imaging was performed for this. On examination, there is a large bulky swelling of the scalp involving the parietooccipital region (left > right) and the left side of the neck with overlying hair (Figure 1). A vaguely palpable defect of the skull is present along the superior and lateral margins of the swelling. There are cutaneous stigmata of NF-1 in the form of multiple café-au-lait macules on the chest, abdomen, and back, along with a few small cutaneous neurofibromas over the abdomen (Figure 2). Neurological examination is otherwise normal. The patient has no other comorbid conditions. Family history is unremarkable; no signs of NF-1 stigmata could be identified in family members. The patient met at least three criteria for the diagnosis of NF-1. A plan was made for debulking surgery of the large plexiform neurofibroma on the scalp/neck, and the patient was referred to the radiology department for preoperative imaging.

**Figure 3:**
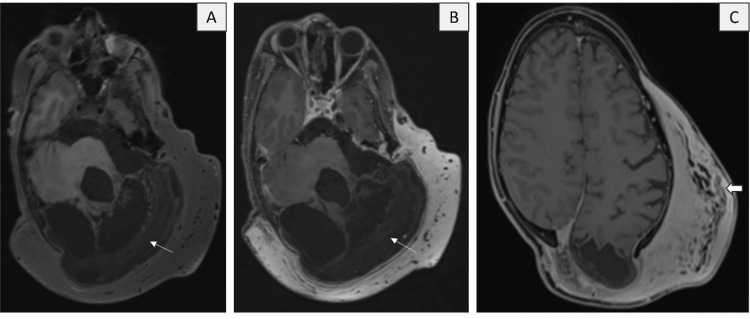
(A) Axial T1 fat suppressed (FS) pre-contrast, (B) Axial T1 FS post-contrast, and (C) Post-contrast volume interpolated breath-hold examination (VIBE) show large, sheet-like homogeneous enhancing soft tissue involving the scalp over the left fronto-parieto-temporal region and bilateral occipital region. A few areas display a linear serpiginous network of fasciculo-nodular lesions (solid white arrow), with intermixed fibrofatty tissue, which represent cutaneous neurofibromas. Thin white arrows indicate gliotic areas in the left cerebellum.

**Figure 4:**
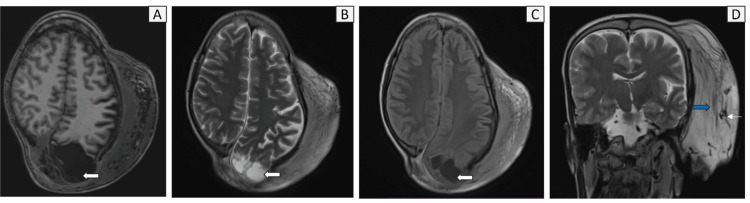
(A) Axial T1, (B) Axial T2, (C) Axial fluid-attenuated inversion recovery (FLAIR), (D) Coronal T2 shows T1 hypointense and T2/FLAIR hyperintense signals with multiple clustered linear fasciculo-nodular lesions (blue solid arrow) and prominent, serpiginous T2 hypointense flow voids (thin white arrow) representing vascular channels present within the lesion. There is a frank defect in the left occipital bone with a herniated meningocele (represented by solid white arrows).

**Figure 5:**
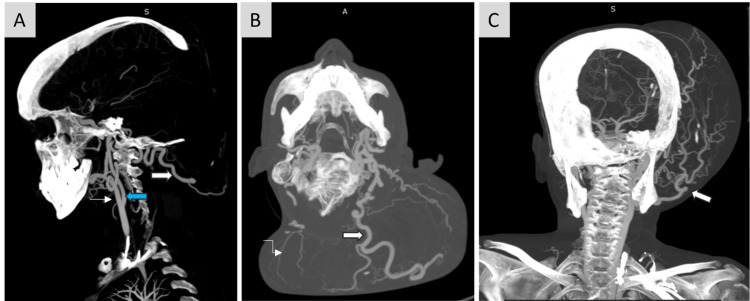
(A) Sagittal, (B) Axial, (C) Coronal maximum intensity projection (MIP) images of CT angiography show dilated tortuous vessels arising predominantly from the terminal maxillary and superficial temporal branches of left ECA (white solid arrow) and a few from the right ECA (curved white arrow) which are seen traversing into and supplying the overlying plexiform neurofibroma. ICA - Blue solid arrow, ECA - White arrow. ICA: internal carotid artery, ECA: external carotid artery

**Figure 6:**
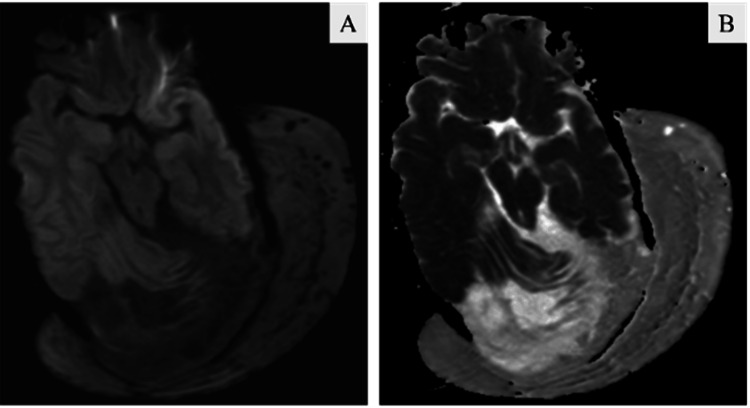
(A) Diffusion-weighted imaging (DWI), (B) Apparent diffusion coefficient (ADC) images show no evidence of any diffusion restriction within this plexiform neurofibroma.

**Figure 7:**
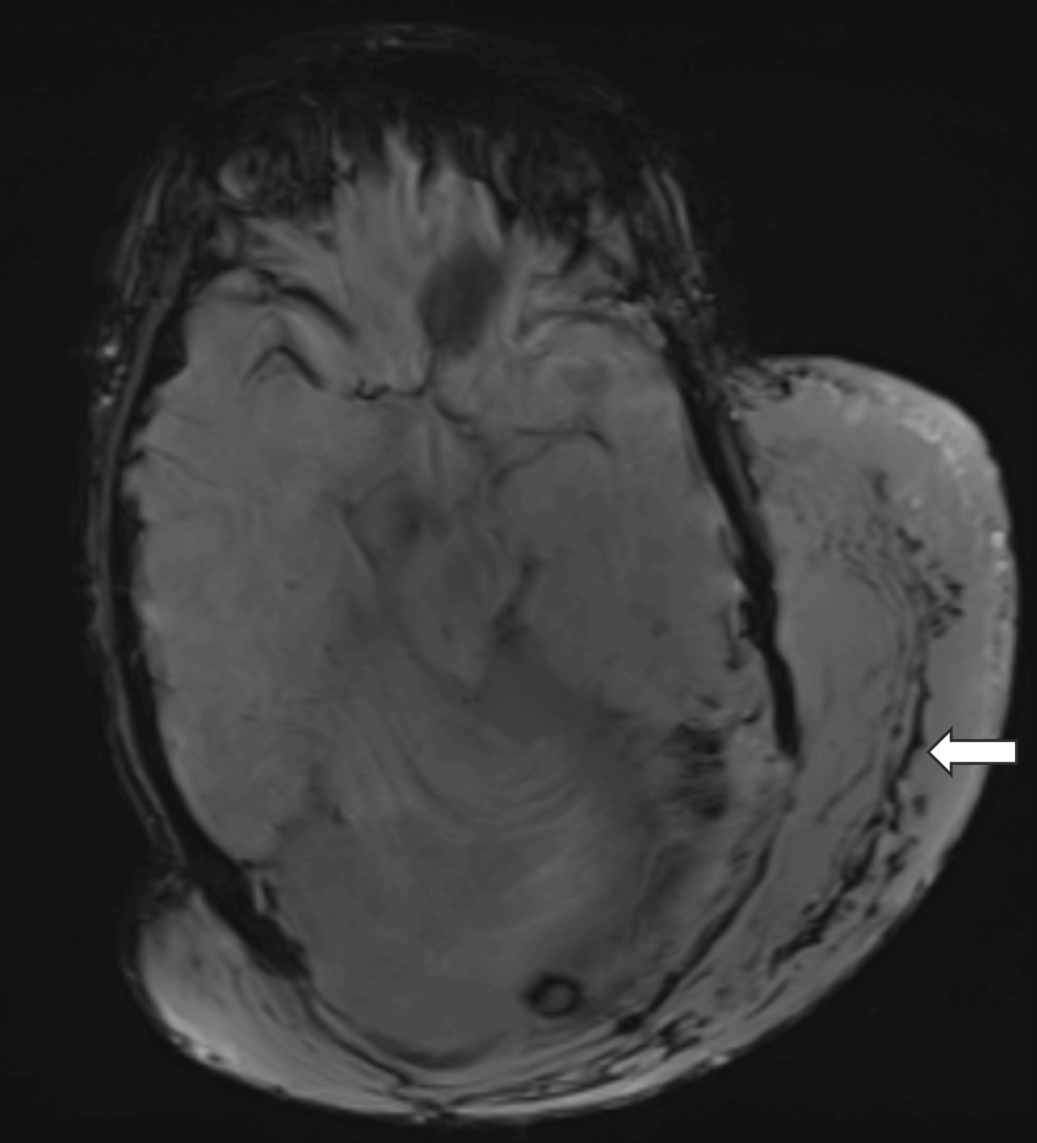
Susceptibility-weighted imaging (SWI) shows multiple curvilinear areas of blooming (solid white arrow) representing the vascular channel.

Contrast MRI revealed a large, bulky homogeneous enhancing sheet-like soft tissue involving the scalp over the left fronto-parieto-temporal region, bilateral occipital region, and the nape of the neck (more on the left side). It showed near-homogeneous T1 hypointense and T2/fluid-attenuated inversion recovery (FLAIR) hyperintense signals. Few areas of serpiginous thickened cutaneous nerve fibers were observed, seen as a "network of fasciculo-nodular lesions" arranged horizontally, with intermixed fibrofatty tissue (Figures 3, 4). A few similar nodular lesions were also noted in the skin over the lesion. Additionally, numerous prominent, tubular, tortuous T2 hypointense flow voids representing vascular channels were present within the lesion, which received feeders from the prominent left external carotid artery (ECA) and the superficial temporal artery (Figures 4D, 5). However, there was no obvious vascular nidus and direct arterio-venous shunting noted, hence the possibility of a vascular malformation was ruled out. On post-contrast sequences, the lesion demonstrated diffuse, near-homogeneous enhancement (Figures 3B, 3C). No evidence of diffusion restriction was noted (Figure 6). Susceptibility-weighted imaging (SWI) shows multiple curvilinear areas of blooming representing the vascular channels (Figure 7).

**Figure 8:**
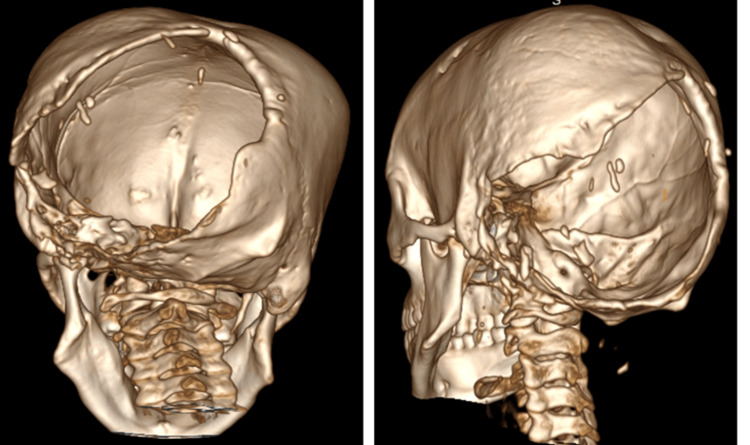
3D volume-rendering technique (VRT) image shows a large defect involving the occipital bone (Left>>Right), involving the lambdoid suture and the mastoid portion of the temporal bone. A normal styloid process is noted on the left side (white arrow); however, it is not visualized on the right side.

On CT correlation, there was dysplasia of the left hemi-cranium and a large defect with scalloped margins involving both sides of the posterior parietal and occipital bones. Superiorly, it extended up to the lambdoid suture (left limb >> right limb). On the left lateral aspect, the defect also involved the mastoid portion of the temporal bone. There was hypo-pneumatization of the left mastoid air cells and significant thinning of the left styloid process (Figure 8). Subtle erosions were seen along the glenoid fossa with mild widening of the left temporomandibular joint (TMJ). There was mild rarefaction and cortical irregularity in the left petrous bone. Additionally, there was marked thinning of the left basiocciput.

This defect caused herniation of a large sac-like structure protruding into the posterior aspect of the soft tissue mass, which contained dysplastic posterior fossa and brainstem structures along with associated ectatic CSF spaces in the left posterior parietal region and posterior fossa. The left cerebellum, including the left tonsil and the inferior half of the vermis, shows near-total loss of volume and architecture, with remnants of dysplastic cerebellar folia seen in the dependent part of the sac. Gliosis and encephalomalacic changes were present in the left parieto-occipital region. Mild herniation of the dilated occipital horn and the surrounding gyri of the parieto-occipital region occurs through the defect. The left thalamus, posterior uncus, midbrain (tectum), pons, and left superior, middle, and inferior cerebellar peduncles were stretched toward the defect, with mild T2/FLAIR hyperintense signals noted in the tectum and colliculus of the midbrain (L>R), as well as in the superior, middle, and inferior cerebellar peduncles.

There was dilation of the fourth ventricle and nearby extra-axial cisternal spaces, some of which herniated through the defect. The posterior thirds of the superior sagittal sinus, left transverse, and sigmoid sinus were visible along the edges of the herniated sac and demonstrate normal flow signals. The left internal jugular vein (IJV) showed reduced caliber with faint signals from the jugular bulb, and a few tortuous flow voids are observed near the left IJV, possibly representing collateral vessels.

**Figure 9:**
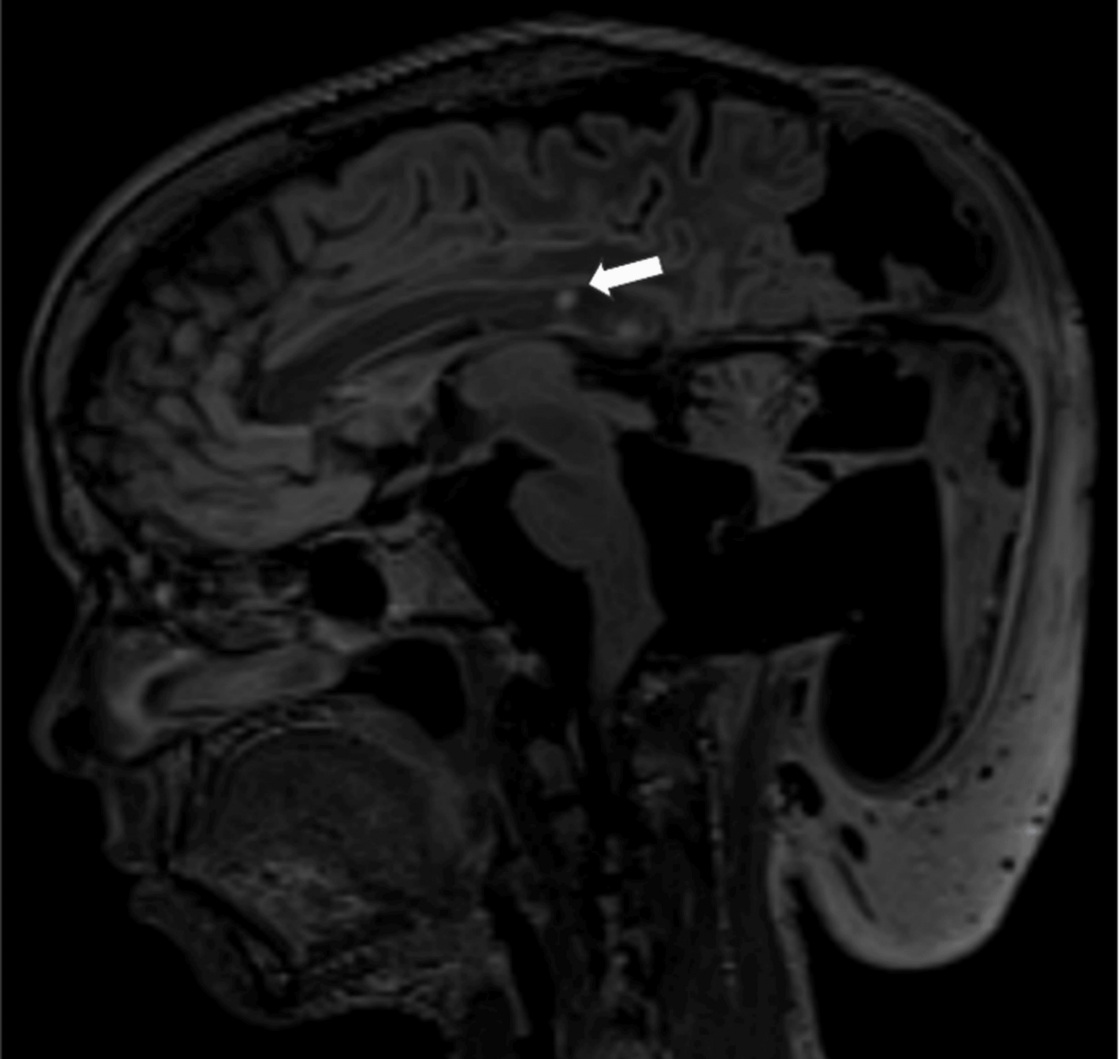
Sagittal fluid-attenuated inversion recovery (FLAIR) images shows focal areas of signal intensities (FASIs) (white solid arrow).

Several relatively well-defined ovoid T2/FLAIR hyperintense and T1 hypointense foci were present in the posterior third of the body and splenium of the corpus callosum. They show no diffusion restriction, blooming, or post-contrast enhancement, which may indicate focal areas of signal intensities (FASIs) in the given clinico-radiological picture (Figure 9).

The patient was scheduled for excision of the plexiform neurofibroma with repair of the posterior encephalocele through duraplasty or cranioplasty. Due to prominent ECA feeder vessels, preoperative embolization was also planned.

**Figure 10:**
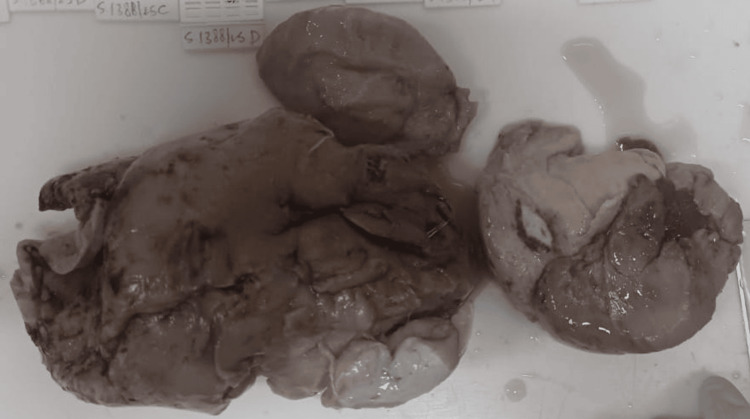
Gross photograph revealed a large, convoluted, bulky mass with grey-white appearance measuring 14.5x11x3cm.

**Figure 11:**
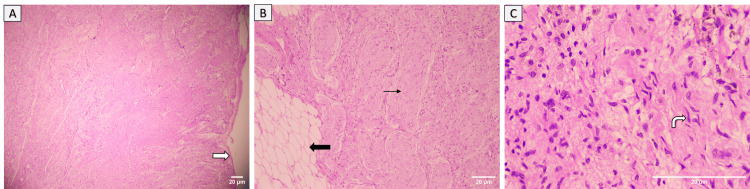
(A) Photomicrograph shows well-encapsulated margin of the lesion (white solid arrow), H&E stain x40, (B) Photomicrograph shows expanded nerve fascicles (black arrow), admixed with fatty tissue (black solid arrow), H&E stain x100, (C) Photomicrograph shows diffuse and nodular proliferation of spindle-shaped cells with wavy buckled nuclei (curved white arrow), H&E stain x400.

Gross cut section of the mass showed a large, convoluted, bulky mass with a grey-white appearance measuring 14.5x11x3cm (Figure 10). Histopathologic examination of samples from the mass revealed diffuse and nodular proliferation of spindle-shaped cells with wavy, buckled nuclei, inconspicuous nucleoli, and moderate eosinophilic cytoplasm mixed with fibro-collagenous areas. Numerous expanded nerve fascicles and focal myxoid regions were observed. The intervening fibroadipose tissue appears unremarkable. The stroma also contains aggregates of neutrophils and lymphocytes forming micro-abscesses, along with areas of hyalinization. These findings were consistent with a neurofibroma (Figure 11).

## Discussion

Neurofibromatosis is a multisystem disorder with a wide range of clinical features. The musculoskeletal signs include scoliosis, posterior vertebral scalloping, short stature, various bone dysplasias, macrocephaly, and multiple non-ossifying fibromas [1,2]. Common bone dysplasias in NF-1 include anterolateral bowing of the tibia, which can lead to fractures and pseudoarthrosis, as well as calvarial dysplasias, most often affecting the sphenoid wing. Our patient had a large defect in the occipital bone. Calvarial defects are rare in NF-1, with only a few cases reported in the literature. The most common location for these defects is along the lambdoid suture, followed by the parietal and temporal bones, and rarely in the occipital bone. Patients with these defects often remain asymptomatic despite their size, with vague complaints like headache. These defects are frequently associated with a large overlying PNF and, in some cases, accompanied by defects in the posterior arch of the cervical spine.

The origin of these bony defects remains unclear, with many hypotheses described in the literature. One suggests abnormal bone development caused by a deficiency of the neurofibromin protein. Previous studies have connected NF-1 loss to increased RAS-GTP levels, which stimulate osteoblast proliferation and hinder differentiation [3]. Additionally, heightened RAS activity has been linked to increased osteolytic activity and bone resorption. This results in progressive ectodermal and mesodermal dysplasia, producing deficient and dysplastic bone. In calvarial bones, this can lead to brain herniation and exposure to external stress, increasing the risk of infection. This explanation accounts for calvarial defects occurring without overlying large PNFs [4,5].

The other hypothesis attributes the condition to prolonged external stress caused by large overlying plexiform neurofibromas. As these tumors grow in size, the pressure they exert on the underlying bone may cause bone remodeling. Over time, this results in the expansion of the defect, which is further worsened by pressure transmitted from CSF pulsations, especially at the sutures [4,6,7].

The CNS manifestations of NF-1 include FASI, optic pathway gliomas, non-optic pathway intracranial gliomas, dural ectasias and meningoceles, and nerve sheath tumors such as plexiform neurofibromas. Our case involved a large PNF over the occipital and neck regions, along with an underlying meningoencephalocele and FASI in the corpus callosum. Malignant peripheral nerve sheath tumors (PNSTs) are highly aggressive soft-tissue sarcomas that can develop from any neurofibroma. They can invade surrounding soft tissue, cause infiltrative edema, and are often found in the extremities and trunk. Patients may present with persistent pain, rapid growth, a new hard texture, or new or unexplained neurologic deficits related to a neurofibroma. There are two forms of PNF: the diffuse form, with margins that are not well demarcated, and the nodular form, which is well circumscribed. Dural ectasia and meningoceles occur due to weakness in the overlying bone [5]. They are typically seen along the course of spinal nerve roots and the posterior vertebral arch. In cases with calvarial defects, the brain tissue within the meningocele is usually dysplastic and gliotic, as observed in our case.

Imaging is crucial in identifying the range of systemic manifestations and in ensuring timely management. CT accurately shows the size and location of bony defects. Contrast MRI is the preferred modality for imaging and characterizing PNFs, especially for detecting malignant transformation. On MRI, malignant degeneration is indicated by heterogeneous signal intensity on T1-weighted images. Key imaging features that help distinguish malignant PNST from benign neurofibroma include a large mass size, peripheral enhancement, a peri-lesional edema-like zone, and intratumor cystic changes. Additional signs that suggest malignant PNST include ill-defined margins, intratumor lobulation, and nearby bone destruction [2].

Managing calvarial defects and PNFs is complex and requires careful consideration of multiple factors, especially in children. The preferred option is dura/cranioplasty; however, repairing bone defects with grafts or titanium prostheses is complicated and often results in poor outcomes, frequently failing due to weak surrounding bone [1,3,5].

## Conclusions

This case highlights a rare calvarial manifestation of NF-1 presenting as a large occipital-lambdoid defect linked to plexiform neurofibroma and meningoencephalocele. Recognizing such unusual skeletal involvement is crucial for accurate diagnosis, pre-operative planning, and multidisciplinary management. Awareness of these uncommon presentations adds to the growing literature on calvarial dysplasias in NF-1.
